# King's College Hospital

**Published:** 1903-09-26

**Authors:** 


### KING'S COLLEGE HOSPITAL.

### A MEMORIAL PICTURE.

The strong probability that before very long King's College Hospital will no longer occupy its present site in Lincoln's Inn Fields, renders the picture which Messrs. Benyon, of Cheltenham, have published of exceptional interest, and it will doubtless in time become quite valuable. The engraving forms a composite picture comprising eight views of the hospital, and the portraits of 20 distinguished physicians and surgeons attached to the institution in past times until the present. Artist first-proof impressions are to be subscribed for at £2 2s. each, and proof impressions at 31s. 6d.

